# Fathering Practices in Sweden During the COVID-19: Experiences of Syrian Refugee Fathers

**DOI:** 10.3389/fsoc.2021.721881

**Published:** 2021-08-11

**Authors:** Therése Wissö, Margareta Bäck-Wiklund

**Affiliations:** Department of Social Work, University of Gothenburg, Gothenburg, Sweden

**Keywords:** COVID-19, ethnographic approach, fathering practices, lockdown measures, Syrian refugees in Sweden

## Abstract

This article explores fathering practices among Syrian refugee families in Sweden. Syrian refugees provide an example of people who migrated because of a single major event: the war in Syria. The article examines the impact of the COVID-19 pandemic on fathering practices. The Swedish COVID-19 strategy differed from those adopted in many other countries. Lockdowns were minimal and were not stringently enforced, based on the assumption that individuals would trust the authorities and would take personal responsibility for complying with their guidelines and recommendations. Previous research suggests that migrants and other vulnerable groups were not always well informed about the public policies introduced prior to and during the pandemic. The article draws on empirical data from a wider research project on the family lives of Syrian migrants in Sweden. The authors present their findings from an analysis of eleven ethnographically informed semi-structured interviews, carried out before and during the pandemic, with married fathers who had been living in Sweden for several years. In this article, they focus on three cases representing fathers with varied educational backgrounds and employment histories. These families had in common what are considered by Swedish standards to be overcrowded living conditions; they were forced to accept close family proximity, both physically and emotionally, as they no longer had the supportive networks they were used to in Syria. The three fathers were found to rely more heavily on information provided by the people with whom they were in contact in Sweden than on policies and recommendations from the authorities. These findings confirmed that the previous experiences among refugees of shifting policies regarding migration and integration had lowered their trust in government. They had learnt that they needed to rely on mutual dependency not only between spouses, but also between parents and children.

## Introduction

In early January 2020, China discovered a new Corona virus among people who had visited a market in the city of Wuhan. On 31 January, the first case of COVID-19 was reported in Sweden. As thousands of Swedes returned home from ski vacations in Italy and Austria, more and more cases were detected. In March 2020, the Swedish Public Health Agency introduced recommendations and general guidelines to reduce the spread of COVID-19 based on the principles of individual responsibility, trust in the authorities and voluntary compliance with government rules.

Despite minimal lockdowns in Sweden, the restrictions had negative effects on the psychological well-being of the individuals since they were unable to socialise as they did before pandemic (Bergnehr et al., 2021). This article contributes to the debate by exploring how refugee fathers living in disadvantaged neighbourhoods in Sweden experienced their fathering practices under COVID-19, and by asking whether these practices have changed their family lives and, if so, how. National statistics show an overrepresentation of foreign-born individuals among COVID-19 deaths and infections in Sweden, and it has been argued that immigrants in vulnerable and marginalised areas often did not understand the Swedish news and advice regarding COVID-19 guidelines (Lager et al., 2020; Public Health Agency, 2021; Rambaree and Nässén, 2020). Other scholars have stressed that it is necessary to adopt a poverty-aware perspective on the effects of COVID 19 since poverty may increase vulnerability to the disease due to its associations with other health conditions, overcrowded housing and difficulties in adhering to social distancing advice (Chang et al., 2021; Krumer-Nevo and Rafaeli, 2021). Swedish data reveal that working class individuals were more exposed to the virus at work, compared to white collar employees (Swedish Trade Union Confederation, 2021).

The empirical data presented in the article are drawn from a comparative study that examined fathering experiences and everyday practices among relocated Syrian migrant families resident in Sweden and the United Kingdom. The original aim of the study was not to examine responses to COVID-19 since the pandemic had not broken out in Europe when the research project was launched in 2019. However, the project’s longitudinal design made it possible to identify the effects of the pandemic since the first wave of interviews was conducted before the pandemic and the second wave during the pandemic. This article focuses on the Swedish part of the study. Although the two studies were conducted in parallel using the same methodology, the extension of the time frame to the end of 2021 meant that the two full datasets were not ready for comparative analysis when this article was being drafted.

## The Swedish Context

The Public Health Agency’s strategy in Sweden to prevent the spread of the Corona virus depended on Sweden’s image as a homogeneous, egalitarian society. The government’s pandemic strategy was underpinned by assumptions about individual responsibility focusing on social distancing, working from home where possible, avoiding public transport, and voluntary isolation for those showing symptoms of the disease. Ethnic segregation and growing social inequality in Sweden were not taken into account. It soon became evident that Sweden was reporting higher numbers of cases and deaths than in neighbouring countries: Denmark, Finland, and Estonia (Ludvigsson, 2020). During the second wave of the pandemic in autumn 2020, supplementary measures were implemented. Facemasks were recommended on public transport, a new pandemic law was passed, and strict and binding rules were implemented regarding public events and gatherings. For example, only small groups were allowed access to restaurants, theatres and sports arenas. However, individuals were left to decide whether they followed the recommendations by staying at home if they had any COVID-19 symptoms, keeping at a safe distance from other people and working from home when possible.

Sweden’s population was estimated to have reached 10,327,589 inhabitants in December 2020, of which 20 percent were born outside Sweden. Between 2014 and 2018, the most common nationality among migrants to Sweden was Syrian (SCB, 2020a). Syrian migrants in Sweden offer an example of people who migrated because of a single major event: the war in Syria. Their arrival in a European country therefore provides an opportunity to explore family practices of one group following forced migration.

Sweden has long operated a “dual-earner, dual-carer family policy” (Björnberg, 1997) with generous state support for working parents, extensive paid parental leave, public childcare and paid leave to care for sick children. Women’s and men’s engagement in paid work is almost equal. Entry into the labour market depends on high educational qualifications and proficiency in the Swedish language, which means that poorly educated refugees who arrived recently in Sweden are excluded from the labour market. The year before the COVID-19 pandemic outbreak, unemployment declined for Swedish-born workers from 4.1 to 3.8 percent, and for those born outside Sweden from 21 to 19 percent. At the end of 2020 when the pandemic hit the labour market hard, young people and those born outside Sweden were worst affected by unemployment (SCB, 2020b). Population growth has also led to overcrowded living conditions. In 2020, 23 percent of those born outside Sweden were living in overcrowded conditions, according to Swedish housing standards (SCB, 2020c).

## Migrant Parenting

An international review of 138 qualitative studies (conducted between 2006 and 2017) regarding parenthood experiences of refugees, asylum-seekers and undocumented migrants revealed that, in almost all the studies, migrant parents described experiences of having to manage different languages, traditions, beliefs and practices, including navigating new systems of education, healthcare and welfare services (Merry et al., 2017). Migration was also associated with changes in family structures, which led to changing roles and relationships. Sometimes role changes caused tension within families, and it was suggested that fathers struggled more than mothers with shifts in gender roles and social expectations.

Research into fathers and fatherhood has burgeoned in recent years, but significant gaps persist (Goldman and Burgess, 2017). Fatherhood and fathering are not homogeneous and encompass economic social, relational, genetic and gendered practices (Brannen et al., 2011; Ives, 2018). To understand migrant fatherhood, it is necessary to consider norms and practices in the new host country as well as in the country of origin, and also the father’s socio-economic status (Liversage, 2015). Migration studies suggest that fathers reinterpret their responsibilities in a new societal context by increasing their share of household duties and childcare (Bergnehr, 2019; Kilkey et al., 2013, Santero and Naldini, 2020; Wojnicka and Pustułka, 2017). According to Breidahl and Larsen (2016), migrants seem to adapt to the host country’s work–family orientation, and that adaption increases with time. In her longitudinal study of refugee men from Middle Eastern countries, Bergnehr (2020) explored narratives about how the men adapted their fathering to new circumstances in Sweden. The study showed that fathers changed their fathering practices, and that they developed new practices as a result of their being in close proximity to their children and wives.

However, resettlement can feel disempowering for families; they experience involuntary separation from their kin and are isolated in dispersed communities (Shapiro and Montgomery, 2020). A Danish study indicated that migrant fathers, being unable to support their families in the new country, faced the risk of double exclusion: both from family and society (Liversage, 2015). A qualitative study conducted in Sweden show how male migrants manage these competing demands as they engage in care work in a nursing home (Storm, 2018). The labour market restrictions encountered by these men in Sweden, together with normative paid work expectations, made them aware of care work as a suitable form of paid employment. Migrant fathers often find themselves in a situation where they experience parallel lives in two societies (Levitt, 2004). They have to resolve different, sometimes contradictory, expectations as parents, at the same time as they try to balance both resettlement and exclusion.

## Theoretical Perspectives

In exploring and analytically grasping the complex processes experienced by fathers as they navigate and negotiate the rebuilding of their everyday lives, this article draws on the concept of family practices (Morgan, 2011). The concept captures the ongoing reproduction, adjustments and development of everyday practices made by migrant fathers in their interactions with their family members and their social networks in the context of Sweden’s public norms concerning “proper” fathering (Larsen et al., 2012). The research project focuses on fathers, but as fathering practices and family practices are closely connected and overlapping, both concepts are used in the analysis.

Research on migrant fathers shows how they include elements from the new society in their everyday lives while also striving to uphold their original main provider role. By taking elements from both new and old experiences in fathering practices, they contribute to the growing number of “hybrid fatherhoods” in Sweden. The family practices approach captures the taken for granted and multidimensional aspects of fatherhood and links history to individual biography (Morgan, 2011). When migrant fathers enter a new country, they do not start from scratch. Rather, through marriage or parenthood, “They come into a set of practices... that are already partially shaped by legal prescriptions, economic constraints and cultural definitions.” (Morgan, 2011, p. 7)

When Morgan (2011) reconsidered the family practices approach, he elaborated on different meanings of practice, such as actions and habits. Thus, Morgan’s conceptual idea about family practices is related to Bourdieu’s writings on practice and habitus. Habitus focuses on the taken-for-granted aspects of everyday family life and the unquestioned routines. In his later writings, Bourdieu (2008) argued that habitus has intentional as well as subconscious elements. However, there are circumstances that require a higher degree of agency when individuals need to plan and take action to handle a situation. Consequently, the practice approach raises questions about the relationship between agency and structure. Morgan (2011, p. 66) recognises that his approach to family practices to some extent seems to over-emphasise agency at the expense of structure. This article elaborates on Morgan’s idea, since it is relevant to the analysis of family practices after migration. Previous research about migrants’ parenting practices has used the concept of adaptation (see for example Bergnehr, 2020; Liversage, 2015; Mussino and Duvander, 2016). Adaptation could be seen as social action, similar to rational choice theories, as a strategy to meet new circumstances and demands. However, research also stresses that adaptation has a price, especially for migrants and others who are expected to adapt to fit the host country’s norms.

## Methodology

The data were collected in ethnographically informed interviews with eleven married couples with children. Inclusion criteria included being a cohabiting partner with at least one child in the age group 3–18. As the study was designed to explore a “later period” of migration, families were selected who had lived in Sweden for at least 3 years. By this time, migrants are expected to have completed resettlement and to be integrated into Swedish society. Services available to new migrants are then reduced or withdrawn.

The first interview was conducted before the pandemic and the second 8–12 months later during the pandemic (September/October 2020). Participants were allowed to choose the location for the interview: for example, a conference room at the university, a public space, or in their own homes. All participants chose to be interviewed at home. An interview guide was used with questions about family and parenting practices in the past, present and future. The participants were informed that they could choose not to answer any of the questions, or parts of questions. In the first round of interviews, the participants were asked an open question: Could you tell me how you came to Sweden? They gave detailed accounts of their situations in Syria during the war, the decision to flee, and the long and dangerous journey to and through Europe. In the interview, the participants were allowed to decide how much they wanted to reveal about these experiences.

The study was given ethics approval by the Regional Research Ethics Committee in Sweden and was carried out with respect for the participants’ dignity without jeopardising their health, security and personal integrity.

The design of the study was informed by previous work with refugees and the researchers’ experiences of conducting research on sensitive issues and/or “vulnerable” populations. The interviews were conducted by one Swedish/English speaking researcher and a research assistant/translator who was an Arabic, English and Swedish speaker. Both interviewers had a background as qualified social workers with experience of working with migrant families. The research assistant had come to Sweden as a refugee from Syria and shared similar experiences to those of the participants. Her experiences proved to be vital in the preparation, execution and analysis of the interviews.

All participants were provided with an information letter in three languages: Arabic, English and Swedish. Participants were also informed about the study orally. As scholars who have engaged in research with refugees have stressed, the process of asking for consent cannot be reduced to a formal protocol. People in vulnerable life circumstances may be desperate for assistance and, therefore, agree to participate in the hope that researchers can help them. However, the participants in the present study were less vulnerable compared to those in camps or to asylum seekers. They were repeatedly reminded that they could withdraw from the interviews at any time without giving a reason and, in such cases, they were assured that their earlier interview material would be withdrawn from the dataset. The participants were informed that all data would be anonymised, stored and used according to the data management policy. They were assured that the data would not be shared with others and that the research project was not connected to the Swedish authorities.

The findings reported in this article are based on a small sample of migrant families from Syria; the results and analysis cannot be generalised to Syrian migrants and their fathering practices more generally. Similarities were found, however, between our findings and those of other studies of migrant fathers (Bergnehr, 2020; Ellingsaeter et al., 2020). In our study, the power to interpret and analyse was in the hands of the researchers, but the research assistant/interpreter drew valuable insights from the interview data. To ensure the accuracy of our interpretations, the fathers were interviewed both separately and together with their wives and children. It was always attend the fathers’ choice to invite family members to interview. It is possible that the fact that the presence of other family members limited the fathers’ accounts, for example, by excluding negative aspects of fatherhood and family life. More positively, the family interviews were found to enrich the material as family members could argue about and discuss different aspects of family life and fathering practices.

As shown in Table 1, the fathers in the eleven families had varied educational backgrounds and employment histories. All but one had been resident in Sweden for 4–5 years and had residence permits. One father had a temporary residence permit since he had come to Sweden 1 year later than the rest of his family, after Sweden had introduced stricter migrant legislation. The eleven fathers were recruited in part by snowball sampling, drawing on the research assistant’s network and knowledge of the Syrian population in the Gothenburg and Halland regions. Another method of recruitment was through an NGO in Gothenburg, working with migrant parents and their children.

**TABLE 1:** Participants’ educational background and employment situation in Sweden.

Pseudonym	Education	Employment in Sweden	Children’s ages	Wife’s education	Wife’s employment in Sweden
Yussuf	Elementary school	Language school	6, 8, 11, 15	Elementary school	Language school
Hassan	University	Taxi driver	5, 10, 12, 17	University	Shop assistant
Khalil	Upper secondary	Unemployed	3, 6, 8	Elementary school	Language school
Adnan	University	Care worker	2, 4, 7, 9	University	Student
Bilal	Upper secondary school	Upper secondary school	4, 6, 8	Upper secondary school	Internship
Salim	Upper secondary	Language school	7, 9, 12	Elementary school	Attending language school
Tariq	University	Unemployed	4, 8, 12	University	Student/care worker
Ali	University	Internship/language school	5, 10	University	Care worker
Bahi	University	Unemployed	1, 3, 8, 12	University	Parental leave
Dabir	University	Shop assistant	1, 4, 8	University	Parental leave
Habib	Elementary school	Factory worker/restaurant	4, 12, 16, 16	Not completed elementary school	Attending language school/cleaner

## The Three Cases

The findings are presented using interview extracts and field notes from three fathers and their families: Adnan, Tariq, and Habib. All names are pseudonyms, and some case information has been changed to preserve the participants’ anonymity. The three cases represent the diversity of the total sample of eleven fathers in terms of education and employment. They had varied connections to the Swedish labour market, and they expressed varied feelings and experiences of being a father in Sweden. The analysis focuses on how being a father changed with the move from Syria to Sweden, fathering during COVID-19, and fathers’ experiences of the authorities’ COVID-19 policies and restrictions. Despite variations in their backgrounds, it was clear from the outset that all three fathers had some factors in common. Being a father was described as the main reason they left Syria since they wanted to give their children a safe childhood. Fatherhood was connected to their breadwinner role: to provide for the family economically and to be the symbolic “head of the household”. The interviewees recounted how their fathering practices had changed after relocating to Sweden since they now spent more time with their children and were more active in childrearing. For example, new fathering practices involved taking children to preschool. In Syria, the younger children stayed at home with their mothers during the day, but in Sweden all children are offered a place in public preschools, and 85 percent of children aged 1–5, born to Swedish parents, go to preschool. The figure for children with migrant parents is 81.7 percent (Skolverket, 2019).

### Adnan

Adnan is married with four children aged 2–9. The two oldest children were born in Syria, and the youngest two in Sweden. The family live in a rented four-bedroom apartment in a small village in the south-west of Sweden. Adnan has a university degree in economics from his home country, where he worked in a private company. In Sweden Adnan found a job at a leisure centre for children aged 6–9, connected to a primary school. The work started as an internship connected to language training that turned into a part-time job. At the time of the interview, Adnan’s wife was still in language school. She was trained as a schoolteacher in her home country but had not found employment in Sweden. She was on parental leave for several years and was following Swedish language courses, hoping to find employment as a pedagogue.

#### How Being a Father Changed From Syria to Sweden

This case represents a family with highly educated parents, and even though the father had to change his career plans and take a less qualified job, he was established in the labour market.

[In Syria] I worked long days, left early in the morning and came home late, and I travelled over the country because my company had sites in different cities. But I never felt bad about it. I knew that my wife and kids were taken care of. They had my parents, her parents, her sisters, you are never alone in Syria. But here we are on our own. It is just our small family, she doesn’t have our relatives here … so we have to work together, I need to help her at…. We only had two children in Syria, now we have four. It is a blessing but it is also a lot to do [laughing]. (Adnan)

The expression “we are on our own” suggests a more intense family life in Sweden, without access to the extended family. Adnan had to compensate for the absence of grandparents, aunts and other relatives. He seemed happy to work with children at the leisure centre, even though this was very different from his job as an economist in Syria. He was glad to have a good relationship with his employment officer, provided as part of the integration programme, who he thought had listened to him and helped him to find a job. In line with Storm’s (2018) findings, moving to Sweden had made care work an acceptable form of paid employment.

#### Fathering During the Pandemic

During spring 2020, Sweden stood out as one of few countries where schools and preschools were open for children aged 1–15 years. The authorities’ main arguments were that children are assumed to be less contagious than adults, and that children benefit socially and mentally from going to school. Furthermore, it was argued that Sweden has a high percentage of dual-worker families and that important functions in society would be harmed if parents had to stay home to look after children. For Adnan and his family, everyday life under COVID-19 went on almost as normal. His wife was studying from home, but the children were still allowed to go to preschool and school, and he went to work. Adnan drove the children to school and preschool in the morning and picked them up in the afternoon.

#### Fathers’ Experiences of COVID-19 Policies and Restrictions

Recommendations from the Public Health Agency varied during the COVID-19 pandemic. At times, national guidelines were supplemented with regional advice. The Swedish authorities had press briefings several days a week; in some periods the briefings were daily. None of the families interviewed reported that they watched or listened to these press conferences. Most of them watched international news programmes, like CNN or Arabic news programmes. However, Adnan was employed by the municipality and worked with teachers and leisure pedagogues, which meant that he was well informed about routines and guidelines, since his job was to tell children and their parents what to do, and what not to do. He complied with the recommendations and thought it was good that schools and leisure centres for children were open. He was not afraid of being infected by the children he worked with, since he trusted the authorities’ message that children were less contagious, but he tried to keep a distance from other parents when they came to pick up their children. Adnan and his wife did not wear masks when they went shopping, and he argued that the authorities did not recommend their use:

I am not an expert. I am no doctor. I am an economist you know [laughing]. I think we need to trust the authorities. They know what is best for the Swedish people. (Adnan)

### Tariq

Tariq lives with his family in the suburb of a large city, in a two-bedroom apartment. They have three children aged 4–12, the youngest was born in Sweden. Like Adnan, Tariq has a university degree and had worked as an engineer in many countries for his company. In contrast to Adnan, he had not experienced personal support from his employment officer in Sweden and was unemployed at the time of the interview. He had taken all the required language courses and had done internships, but without finding in a job. Tariq’s wife has a university degree and was taking a university course in Sweden. She was also working part-time at a residential home for older people. This case represents a family where the father is unemployed, in contrast to the mother, which causes frictions in the family.

#### How Being a Father Changed From Syria to Sweden

Tariq expressed ambiguities and ambivalences regarding his changing role as a father in Sweden. He spent more time at home and with his children but, in stark contrast to Adnan, he was very unhappy with his situation as he was unemployed and could not practice his profession. His wife had been more successful in finding a job, and so could contribute to family income. For Tariq, relocating to Sweden implied a loss of his professional identity as an engineer, and he expressed concerns about his exclusion from the Swedish labour market.

I was working at construction sites in other countries before I came to Sweden. I have been working with people from the United States, from Germany, from Egypt, from all over the world. I have competence. But here, Sweden doesn’t trust my competence. They say they have different standards and they can’t give me a job. I am laughing and crying at the same time. Why is my competence enough in other countries but not here? (Tariq)

This quote affords an insight into what constitutes masculine competency. Tariq’s loss of professional identity was not compensated by his new fathering role. He said he participated in activities with his children with the comment: “This is what I do, but I want to work.”

#### Fathering During the Pandemic

Tariq had younger children who went to school; their activities were much the same as usual. Tariq had contact with an NGO working with migrant fathers and their children, which continued to arrange some activities for fathers and their children; for example, he and one of his sons participated in a swimming course.

We have been able to participate, my children like it, and it was really good for me to meet with other dads. Since I can’t go to the mosque this was good. And I think that my children get to know me more, they turn to me more now, you know at home, it’s like I am more important. (Tariq)

The reason why Tariq could not go to the mosque was because of the restrictions on public assemblies, including gatherings for religious practice. This quote shows that Tariq is trying to find a new role and place for himself. His major concern was about the pandemic’s effect on the family economy, and more specifically about his prospects of finding a job. He expressed a growing mistrust of Swedish employers and labour market institutions and the way that integration policies are implemented in practice. In contrast to Adnan, Tariq talks about problems in the context of Swedish society and the importance of staying in contact with fellow Syrian men in Sweden.

#### Fathers’ Experiences of COVID-19 Policies and Restrictions

Tariq argued that Swedish authorities are unclear about what you “should do” and what you “have to do”. Unlike Adnan who had developed trust in Swedish authorities, Tariq had experienced stressful encounters with teachers who argued that their child should stay at home because of symptoms, but he and his wife argued that the child was healthy enough to be in school. Furthermore, he expressed an ambivalent attitude towards the Swedish authorities’ ability to implement “good policies”, both in relation to integration but also in relation to the COVID-19 strategy. He was glad that children were allowed to go to school and was frustrated about restrictions limiting his own social life. Tariq was recruited to the research project through an NGO working with migrant fathers. The NGO employed “cultural interpreters” with the aim of helping and guiding migrant fathers in their contact with Swedish society. Cultural interpreters have migrant backgrounds but have lived in Sweden for a longer period. Tariq argued that the cultural interpreter was helpful in relation to the COVID-19 pandemic:

We speak the same language but he has more information, he knows the latest news. And you hear a lot, you know, there are rumours about this disease and about the vaccine. So it is good for me to speak to him, and then I can tell my family and friends: this is what we should do. (Tariq)

### Habib

Habib is married with four children, aged 4 and 12, and twins aged 16. The three oldest children were born in Syria and the youngest in Sweden. He had had his own hairdressing salon in Syria. Unlike Adnan and Tariq, he has no higher education. In Sweden, when he was interviewed, he was working full time in a small factory and extra hours at a restaurant. The family lives in a three-bedroom apartment in a small town. His wife does not have higher education from her home country. She was almost illiterate when she came to Sweden and has difficulties in reading and writing in Swedish. However, she managed to get a job as a cleaner and so contributes to the family income. This case represents a family who have been integrated into the labour market despite having little formal education.

#### How Being a Father Changed From Syria to Sweden

Habib’s work−family life was different in Sweden compared to Syria. In Syria, he owned his own business and worked long hours, but he could decide how much and when he wanted to work. They received a lot of help with childcare from their extended family, so Habib did not have to engage in caring activities. Even though he was managing two jobs in Sweden, he spent more time with his family than in Syria. His wife was attending a language school and had recently found a job, which meant that she left home early in the morning, and Habib was in charge of getting the children ready for school. He thought it was good for his wife to have a job, as it ended her social isolation, and she had more opportunities to learn Swedish. However, he had to help the older children with their studies, something his wife was unable to do. Like Adnan and Tariq, he was more involved in childcare in Sweden but struggled with feeling that he could not live up to what was required. Habib also missed the extended family relations that they had enjoyed in Syria.

#### Fathering During the Pandemic

Habib’s everyday life was more affected by the pandemic than that of Adnan and Tariq. His twins had to study at home. Nor were they allowed to continue with their sporting activities because of the restrictions for children aged 16 and over. Both his daughter and son were dedicated football players, and Habib liked to watch them play.

It is crowded now. They are at home. My wife is at home. They are the same age but are in two different classes. So, they need to have their lessons separately. And I work shifts, so sometimes I need to sleep during the day. I need peace and quiet. It is not possible [laughing] And then my wife picks up the little one from preschool and then it is a crazy house. (Habib)

Habib said that he was allowed to rest in a small changing room at the factory. Unlike Adnan’s and Tariq’s families before the pandemic, Habib and his family used to get help from an older relative, an aunt of his wife. She used to come to stay for a couple of days and help out with cooking, cleaning and taking care of the children. The aunt is aged over 70, and Habib and his family were aware that older people need to be protected from the virus. Thus, she was not visiting them at the time of the second interview. On a positive note, Habib said that, since the oldest children were at home more, they were able to be involved in caring activities. Sometimes his daughter picked up the younger siblings at school, and she also prepared food. During the pandemic, Habib and his daughter prepared meals together.

Habib’s employment at the factory was not affected by the pandemic, but he used to work extra hours at a restaurant as a dishwasher. In Sweden, restaurants remained open during the pandemic, but the number of guests decreased, resulting in layoffs of staff. Since the middle of March 2020, Habib had not been offered work at the restaurant, which had impacted on family income.

My pay at the factory is not very high, but I want to give my family a good life. They are used to a more privileged life in Syria. The loss of the extra money is not a disaster, but it is more difficult for me to send money to my elderly relatives in Syria. (Habib)

#### Fathers’ Experiences of COVID-19 Policies and Restrictions

In Habib’s family, the children were the ones who informed the parents about government guidelines on handwashing and social distancing. His children told him: “we have to wash hands and keep distance”.

Like Tariq, he expressed ambivalent feelings about whether or not they trusted the Swedish authorities and its guidelines and recommendations.

I am thankful to Sweden for receiving me and giving me and my children a safe future. Sweden has very good schools, and people are friendly, but the Swedish authorities can be very confusing. Changing rules and sometimes you feel you have no power. So, I trust Sweden, but they are not always right about things. (Habib)

Further, the interview with Habib illustrated how individual family members responded differently to government guidelines, which led to disputes within the family:

My oldest child [16] says that the authorities say that we should not wear masks, that masks don’t protect against corona. Here you never see people in masks, not in the shops, not on the train. But all over the world you see people with masks, and they have rules and have to wear them. I thought we should buy masks but my daughter said no. She said that she would be ashamed if I walked around with a mask. But, if the government in other countries say you should have mask, why is it different here? It is the same virus. (Habib)

## Discussion and Conclusion

In this article, the authors have explored the fathering practices of Syrian refugees in Sweden during the pandemic, asking whether they have changed and, if so, how. The way that family practices are discussed in the interviews provides valuable insights into how fathering and family are shaped in the interaction between family members.

The study’s longitudinal design, and the unforeseen outbreak of the pandemic, made possible an analysis of the adaptation of Syrian refugees’ fathering and family practices over time. The research was also able to capture the reactions of families as they dealt with the effects of the pandemic. Fatherhood, in the sense of taking responsibility for the family’s standard of living, safety and protection was found to be a driving force for Syrian migrants in Sweden. These responsibilities also served as prerequisites for adjusting their fathering practices in Sweden.

The process of adaptation to new ways of fathering was found to vary among the participating fathers. Adnan had developed new fathering practices and had come to regard care work with children at a leisure centre as a way of providing for the family. He saw his role in his family as “helping his wife”, maintaining that she was the most important person for the children. Adnan’s experiences pointed to a continuum between fathering practices as habits and action. Some practices turned into routines that were carried out more automatically, emphasising the significance of social structures in family practices. The diversity of these reactions exemplifies how individuals face new situations that require planning and action. In Adnan’s case it was also evident that the children played an important role when new practices were being negotiated, since the children instructed their parents about how to behave and handle certain situations.

Tariq was involved in the everyday care of his children, but he regarded the practice as provisional while waiting for a job. His loss of professional status dominated his and his family’s situations. His own family roles and those of his wife had been reversed, and the future had been put on hold. Tariq struggled with his new role in Sweden and was not content with being unemployed and unable to provide for his family. He did not view his fathering practices as self-selected, and, in line with the findings from other work (Shapiro and Montgomery, 2020), his resettling practices could be seen as disempowering. In Tariq’s case, a disparity was found between his fatherhood ideals and his current fathering practices. On the one hand, it could be argued that Tariq felt that the situation in Sweden was out of his control. He realised that the ways he thought about fatherhood were challenged, both in relation to individual and collective habitus. On the other hand, Tariq’s way of holding onto his professional identity and beliefs about his role as a father could be viewed as an expression of agency. Even though he had modified his practices to adapt to changing circumstances in Sweden, he managed to preserve his values. The pandemic reduced Tariq’s opportunities to meet with friends since assemblies in the mosque were not allowed. However, he and his children participated in a “father−child” project run by an NGO, and Tariq appreciated that it contributed to a better relationship with his children.

Habib’s family had maintained a lifestyle with a supportive network. They sent money back home after migrating to Sweden, but the pandemic stopped these remittances since Habib lost his additional income from working in a restaurant. Due to home schooling for the two oldest children, Habib described their home situation as chaotic. They struggled to get through everyday obligations and created ad hoc solutions for developing new practices.

The three fathers and their families were affected differently by the pandemic, since restrictions and guidelines on home schooling varied depending on children’s ages. For all three fathers, the pandemic resulted in a more intense family life, because they spent more time at home with their children. The pandemic demanded actions and pushed the fathers into greater participation in fathering. Already before the pandemic, Adnan and Habib had adjusted their fathering practices, and ways of thinking about fatherhood; their fathering habitus. When Habib’s situation at home changed due to his children’s home schooling and loss of support from an older relative, he took action to resolve the situation. Habib was happy to do more cooking with his 16-year-old daughter who spent more time at home due to physical distancing rules. This intense family life also had negative effects, including difficulties in finding time for rest and recovery. By contrast, changing routines were more challenging for Tariq, who was less convinced by the “new fatherhood” model. He felt that the loss of regular contact with other men at the mosque was difficult to handle. Furthermore, for Tariq, who had been unemployed for several years, the pandemic increased his lack of belief in “the system”. He argued that he would never get a job in Sweden, which caused him much distress.

The research presented in this article contributes to sociological theory about fathering practices by showing clearly how three migrant families in Sweden adapted their parenting roles in response to changing circumstances and societal structures during the pandemic (Bergnehr, 2020; Bergnehr et al., 2021; Liversage, 2015) By providing valuable insights into their perceptions of interactions within these families, the research also contributed to policy development. It revealed how Swedish government policies could be modified to take account of the specific needs of refugee families.

Since the Swedish government and authorities based the COVID-19 strategy on recommendations rather than on prohibitions, the responsibility for following and interpreting guidelines was placed on the individual. Refugees are, however, obliged to follow government rules and regulations concerning their status as migrants. All three fathers claimed difficulty in understanding policies on residence permits, rules for benefits and social insurances; they argued that policies seem to change all the time. This reaction also applied to recommendations and policies regarding the pandemic. The fathers’ previous experiences of changing and inconsistent policies in relation to migration and employment seemed to influence their opinions about the Swedish pandemic strategy and the instructions they received from the authorities. Guidelines about wearing a mask were ambiguous; several of the parents interviewed wore masks when they did their shopping, even though authorities in Sweden had not advised people to do so at the time of the interviews.

The three parents discussed here said that in some matters they trusted the authorities in other countries more than in Sweden. Even though the authorities’ decision to keep schools and preschools open was welcomed by the families, it caused uncertainty, since they were aware that schools and preschools were closed in other countries. However, Adnan felt that he relied on the authorities’ guidelines, referring to the fact that “they are the experts”. Habib was more ambivalent towards the Swedish guidelines, and Tariq clearly conveyed a lack of trust. Tariq maintained that he put more trust in the information he received from people engaged in an NGO with whom he shared the same cultural background and native language.

Families in the three cases had in common their overcrowded living conditions. They were forced to live in close physical and emotional proximity both before and during the pandemic. As migrants, they no longer had the supportive networks they were used to in Syria. Instead, they had to create mutual dependency between parents and children. The pandemic revealed the limits on opportunities for small talk and information exchange between parents and staff in school and daycare centres. The findings suggest that the Swedish authorities need to cooperate more closely with community-based organisations to reach out to migrants and marginalised groups. Face-to-face contact with government representatives was much appreciated when available. In its absence, the families relied more heavily on information provided by the people with whom they had a relationship and could meet on a regular basis.

## Data Availability

The raw data supporting the conclusions of this article will be made available by the authors, without undue reservation.
